# Ten sustainable steps infectious diseases professionals can take to mitigate the climate crisis

**DOI:** 10.1017/ash.2024.394

**Published:** 2024-09-25

**Authors:** Shreya M. Doshi, Pamela Lee, Saul Hymes, Judith A. Guzman-Cottrill, Preeti Jaggi

**Affiliations:** 1 Division of Infectious Diseases, Children’s National Health System, Washington, DC, USA; 2 Department of Pediatrics, School of Medicine, The George Washington University, Washington, DC, USA; 3 Division of Infectious Diseases, Harbor-UCLA Medical Center, Torrance, CA, USA; 4 Albany Medical College, Department of Pediatrics, Division of Infectious Diseases, Albany, NY, USA; 5 Oregon Health and Science University, Department of Pediatrics, Division of Infectious Diseases, Portland, OR, USA; 6 Emory University, Department of Pediatrics, Division of Infectious Diseases, Atlanta, GA, USA

## Abstract

Climate change and pollution harm the public. The healthcare industry disproportionately contributes to greenhouse gas emissions. Infection diseases professionals including infection preventionists and antimicrobial stewards are uniquely positioned to mitigate the environmental impact of our daily practices. We highlight 10 actionable steps that infectious disease professionals can incorporate into daily practices, thereby mitigating the impact of climate change.

Climate change and pollution are leading causes of premature death worldwide. The World Health Organization estimates that 1 in 4 deaths is secondary to preventable environmental causes such as air, water, and soil pollution^1^ and that an additional 250,000 deaths per year occur due to climate change.^2^ The US healthcare sector accounts for 8.5% of its greenhouse gas (GHG) emissions, which causes significant morbidity and mortality. In 2018, healthcare-associated air pollution and emissions were estimated to cause the loss of 388,000 disability-adjusted life years in the United States alone.^3^ In addition, climate change significantly exacerbates global infectious diseases (ID) including emerging and reemerging ID.^4^

The US healthcare sector’s disproportionately large^5^ contribution to global GHG emissions and healthcare waste has spurred substantial interest in healthcare sustainability nationwide.^6–8^ Healthcare sustainability as a field quantifies the unintended environmental impact of healthcare and evaluates care delivery that affirms both planetary and patient health.^9^ Interventions promoting healthcare sustainability have led to significant reductions in healthcare-related GHG emissions^10^ and healthcare waste.^11^

There is a dearth of ID-focused healthcare sustainability currently, even though ID oversees infection prevention and control (IPC), a significant contributor to healthcare waste.^12,13^ The ID community also developed and promotes the field of antimicrobial stewardship, which shares fundamental values of healthcare resource conservation with healthcare sustainability. Given these correlations, ID practitioners (including healthcare epidemiologists, infection preventionists, and antimicrobial stewards) have a unique and valuable opportunity to become leaders within healthcare sustainability. Here, we will provide 10 actionable steps that ID preventionists, researchers, and other ID professionals can undertake to further healthcare sustainability within their professional work.

## Let’s start by throwing the right waste into the right bin

The healthcare industry generates a staggering 5.9 million tons of waste per year in the United States, including 1.7 million tons of plastic waste.^14,15^ Healthcare waste is sorted into multiple disposal streams, including incineration, landfill, recycling, and compost, each of which confers a different environmental impact. Biohazardous waste generates higher GHG emissions compared to non-biohazardous waste because high-energy processes such as autoclaving or incineration are frequently required for disposal. Unfortunately, waste audits have repeatedly demonstrated that non-biohazardous waste is frequently segregated improperly into biohazardous waste bins, resulting in unnecessary environmental harm as well as increased healthcare costs.^16,17^ Biohazardous waste has therefore become a highly targeted area in healthcare sustainability due to its environmental and financial benefits. IPC likewise is a key stakeholder in waste management, particularly in ensuring that potentially infectious waste is appropriately managed.

**Figure 1.**
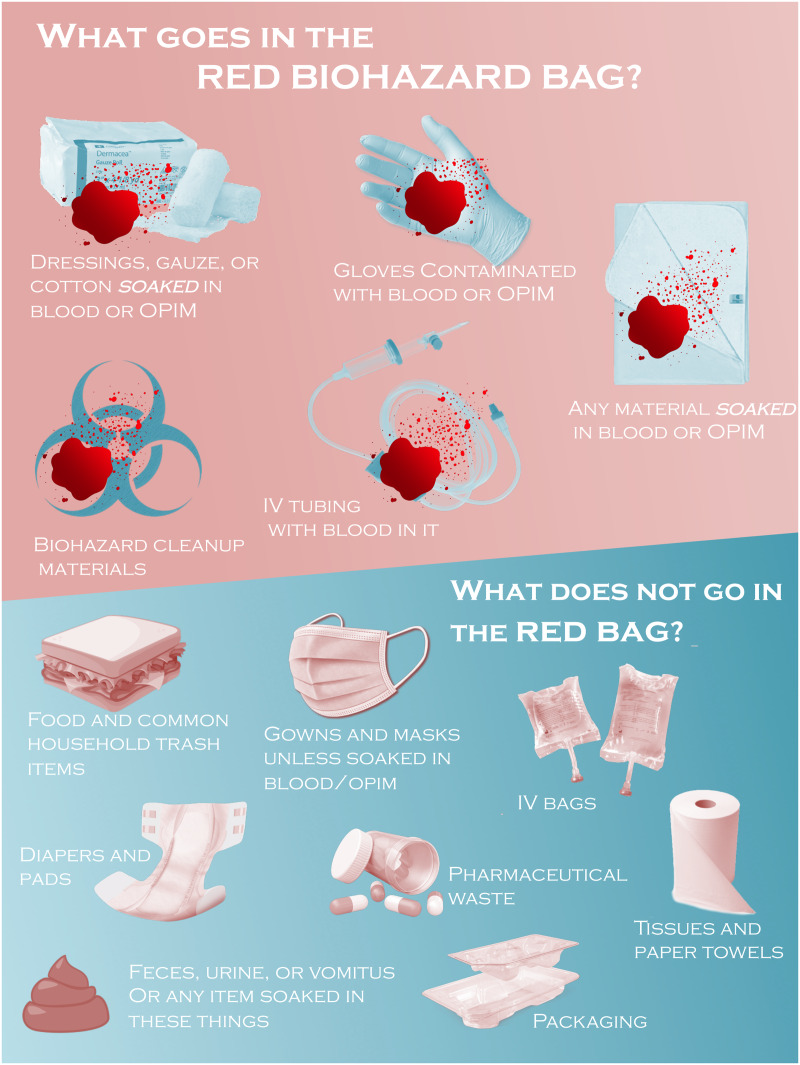
Example red bag waste initiative poster, reprinted with permission from authors. Moretti K, Karb R, Durand R, Kobayashi L, Hayward AS. Wasting No Time: Implementation and the Climate Impact of a Solid Waste Stream Process Intervention in a Large Academic Emergency Department. R I Med J (2013). 2021;104(9):34–37. OPIM: Other Potentially Infectious Material

Effective waste segregation aligns with the priorities of both IPC and healthcare sustainability and is an outstanding opportunity for collaboration between these fields. Methods to improve waste sorting include education, strategically placing biohazardous waste bins so they are not overused, reducing the size of biohazardous waste bins, and ensuring that signage is current and prominently placed near waste bins. Promoting strategies that are user-centered and minimize the additional burden to busy providers is crucial to attain lasting changes to healthcare waste streams (Figure 1).

## Follow “smart” transmission-based isolation (and de-isolation) precautions

Personal protective equipment, or PPE, is a major component of healthcare waste—close to 70% of plastic municipal solid waste collected during a waste audit of an inpatient medical unit was PPE.^18^ Healthcare IPC programs should take time to review all isolation policies, with the specific goal of reducing unnecessary use of PPE during patient care. This includes prompt de-isolation of hospitalized patients when they are no longer deemed contagious and following standard precautions for endemic multidrug-resistant organisms when program surveillance supports this practice change.^19,20^ Healthcare IPC programs should continue to reevaluate the utility of contact precautions for those with a history of antimicrobial-resistant infections.^21^

Equally (or even more) important is to practice judicious or “smart” use of PPE, meaning employing PPE only when truly necessary for patient safety. IPC programs can regularly review internal policies regarding PPE to ensure they are up to date. Furthermore, IPC programs can consider healthcare waste as a factor when implementing or updating policies that require PPE use. Finally, IPC programs should routinely educate their healthcare workforce about their individual roles in preventing unnecessary PPE use for both patient safety and sustainability reasons. Such education can be included in new employee orientation, during hospital IPC rounds, and in annual IPC competency education. There is an opportunity for IPC programs to incorporate education about judicious use of PPE while meeting the Centers for Diseases Control and Prevention (CDC) core practices^22^ which are utilized by healthcare regulatory bodies in the United States, including the Joint Commission.^23^ For a patient on standard precautions, in general, this could also include performing hand hygiene alone rather than gloves for examination of patients when there is no contact with body fluids anticipated.

## Stop wasting so many medical supplies

Many facilities have IPC policies that require the discard of all “potentially contaminated” single-patient room supplies after discharging a patient who was hospitalized under contact isolation precautions. This practice leads to substantial unnecessary waste plus thousands of dollars of healthcare loss.^24^ To minimize this waste, IPC programs can collaborate with inpatient unit leadership to decrease the number of disposable supplies stocked in patient rooms. In addition, techniques such as disinfecting packaged items rather than disposing of them may also be effective in decreasing waste and preventing infections.^24^ Further studies examining how such processes may be implemented widely in healthcare systems, as well as of the IPC and environmental sustainability impacts of these techniques, are necessary.

**Figure 2.**
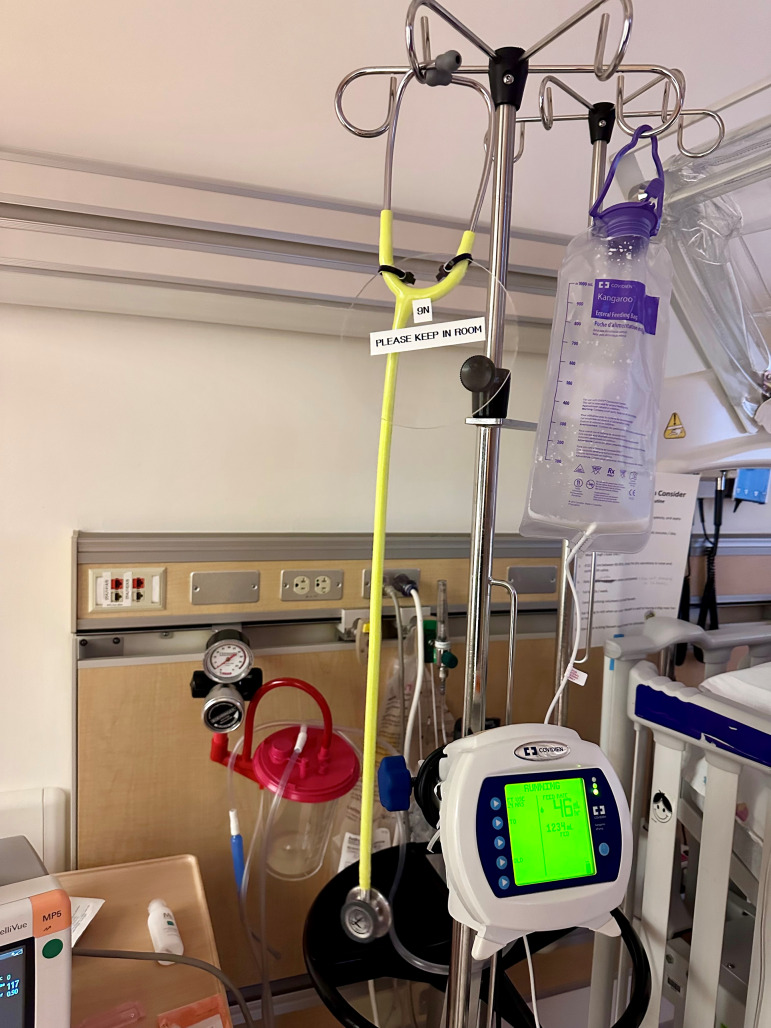
Example of placement of a high-quality stethoscope that was routinely cleaned and disinfected as “high-touch surfaces.”

IPC programs should also collaborate with their facility’s logistics and procurement leadership to find opportunities for minimizing disposable items and replacing them with reusable supplies whenever possible. Examples may include surgical instruments, endoscopes and their accessories, and commonly used items such as “single-patient stethoscopes.” One children’s hospital recently discontinued the purchase of single-patient stethoscopes for patients in isolation precautions. The disposable stethoscopes were considered poor quality by clinicians and needlessly contributed to hospital waste. The solution was to routinely stock 1 high-quality stethoscope at the bedside in every inpatient room (Figure 2). The stethoscopes are routinely cleaned and disinfected as “high-touch surfaces.” As a result, clinicians no longer carry personal stethoscopes, further decreasing the risk of indirect pathogen transmission. A renewed focus on high staff competency for medical supply cleaning and disinfection, rather than relying on disposable items alone, can reduce contributions to the medical waste stream. Discussions about minimizing healthcare waste should be happening from healthcare administration (facility level) to the bedside staff (patient level) and everyone in between.

## Attend a medical meeting or plan an interview…virtually

The American Society of Tropical Medicine and Hygiene analyzed that in 2019, approximately 4,000 attendees collectively traveled approximately 27.7 million miles to reach the conference venue, equating to roughly 58 return trips to the moon for their annual conference alone.^25^ High-priority solutions to reduce the massive GHG emission footprint from transportation to meetings include decentralized conference “hubs,” a rotational model alternating between in-person and virtual formats, as well as hybrid conferences that combine both modalities. One way to increase accessibility to trainees for a professional meeting is to reserve a conference room and invite others who may not typically join to watch lectures with a faculty member or attend. This is a great way to create social connection and expose trainees or other clinicians to ID topics and greatly reduces the environmental impact.

Virtual interviews should be considered as the new standard for screening interviews. The environmental impact of interviewing for residencies by 1 graduating medical student class in the prepandemic era (when this process was conducted in person) was estimated to be approximately 1 million pounds of GHG emissions, which is roughly equivalent to the carbon sequestration capability of a 700-acre forest.^26,27^ The virtual option for ID fellowship was assessed by both fellowship program directors and virtual interviewees in ID and both groups prefer to have a virtual option for recruitment.^28^

## Reduce transportation emissions from patient care, infection prevention, and stewardship work

Telemedicine decreases healthcare GHG emissions and costs and may also decrease motor vehicle-associated injuries and fatalities.^29,30^ The Pediatric Infectious Diseases Telehealth Working Group found that in the pre-COVID era, only 13%–20% of ID providers used telehealth modalities.^31^ However, the same group published data showing that post-COVID, telemedicine utilization increased 4-fold, and satisfaction with telemedicine modalities grew to over 90%.^32^ Further research is needed regarding which patients are best suited for telemedicine, though many report that established patient visits are often easiest as rapport and initial evaluations have already been established.

Infection prevention and antimicrobial stewardship are also able to be practiced remotely and may mitigate limited staffing across the United States.^33^ Early in the coronavirus disease 2019 pandemic, the CDC successfully conducted telephone and video-based infection control assessment and response (TeleICAR) consultations to over 600 nursing homes.^34^ The CDC has created a library of standardized IPC checklists that can be used for both in-person and virtual IPC consultations.^35^ A statewide antibiotic stewardship program in Utah was implemented in 2016 and has successfully provided both clinical and stewardship consultation to 16 small community hospitals.^36^ Similar telehealth stewardship programs have reported successful results, including reduced broad-spectrum antibiotic use and reduced cost.^37,38^

Telehealth has been endorsed by the Infectious Disease Society of America (IDSA) “to provide up-to-date, timely, cost-effective subspecialty care to resource-limited populations.”^39^ Beyond these cited benefits by IDSA, telehealth also decreases negative climate impacts.

## Decrease pharmaceutical waste

From manufacturing to consumption and eventual waste disposal, the climate impact of inpatient pharmaceutical waste, particularly antimicrobial waste, is substantial. Considerable energy is required for transportation and autoclaving or incinerating of pharmaceutical waste, and landfill waste generates methane, a potent greenhouse gas. Unused drugs that are discarded can occur with any weight-based dosing regimen and is a particular problem with pediatric patients. Pediatric doses are often prepared in advance but subsequently discarded if not administered due to patient discharge or cancellation/modification of treatment. Unfortunately, these unused doses cannot typically be repurposed. At least 3 pediatric healthcare systems have separately estimated their antimicrobial waste to cost more than $100,000 annually.^40,41^ It is important to note that this financial estimate does not encompass the additional costs associated with autoclaving, incinerating, transporting, or managing pharmaceutical waste through waste management companies. Pharmaceutical waste has also been documented in adult centers and should be considered for antibiotics such as daptomycin or aminoglycosides due to weight-based dosing.^42^ Strategies to prevent unnecessary waste could include placement of stop times in the electronic medical records at least 24 hours in advance to notify inpatient pharmacy staff. Of note, most of this drug waste has been documented with antimicrobials but is likely occurring with other pharmaceutical classes.

As practitioners in Antimicrobial Stewardship Programs (ASP) and IPC, collaboration with pharmacy departments is imperative to optimize dosing regimens and minimize waste. The good news is that widely accepted antimicrobial stewardship initiatives, such as eliminating prescribing of unnecessary antimicrobials, using shorter durations of therapy when possible, adding stop dates in advance, and advocating for initial oral (PO) drugs when clinically appropriate, all reduce the climate footprint of healthcare and potentially decrease pharmaceutical waste. One of the only lifecycle assessments of an antimicrobial studied is that of vancomycin which revealed that the production of 1 gram of vancomycin requires 0.6 kilograms of raw materials. This includes water for fermentation and irrigation, extraction of dextrose from corn and soy flour, and the consumption of crude oil and natural gas for processing and transportation.^43^ This information may be helpful for ASP teams that are targeting vancomycin waste.

## Petition your healthcare system for more climate action

There is strong support for healthcare sustainability among healthcare workers. In 2023, a US survey of 1001 healthcare workers demonstrated that over 80% of those surveyed believe that healthcare organizations should address climate change.^44^ ID physicians have well-established partnerships with the hospital administration, nurses, and other clinicians through IPC and antimicrobial stewardship work. These relationships make us well suited to lead advocacy efforts for more climate action in partnership with our colleagues. There are several objective, actionable steps that healthcare systems can now take. First is the Health and Human Services (HHS) Climate Pledge for specific measurement and mitigation of GHG emissions. Other options are to apply for the Joint Commission Sustainability Certification or join Practice Greenhealth, an organization that helps hospitals reduce environmental impacts. Finally, several centers now have multidisciplinary healthcare sustainability teams that include clinicians. Healthcare workers in Pittsburgh successfully petitioned their administration to sign the HHS Climate Pledge to reduce GHG emissions and formed a sustainability team with clinician-directed projects to reduce emissions. Another powerful request is to ask your center to divest any financial investments from the fossil fuel industry as suggested by this recent New England Journal of Medicine viewpoint.^45^ If you are interested in some coaching about how to organize with others to affect change, consider applying for the virtual Climate and Health Organizing Fellowship^46^ through Cambridge Health Alliance. Finally, collective action with colleagues can build camaraderie among the healthcare team and help mitigate climate anxiety.^47^

## Promote better food options and decrease food waste in healthcare

Many ID clinicians and antimicrobial stewards already engage in legislative advocacy to modify hospital purchasing programs to procure meat raised without the routine use of antibiotics, and this mitigates both antimicrobial resistance and also GHG emissions. Antibiotics have been shown to alter the structure and activity of mammalian fecal microbiota, which may result in more GHG emissions.^48^

Beyond antimicrobial stewardship in agriculture, reducing the amount of meat consumed would significantly decrease GHG emissions. The climate impact of food can be measured by using tools such as the Coolfood calculator.^49^ By partnering with dietician colleagues in hospitals to promote less meat to be served on hospital menus, ID providers can directly decrease the amount of meat purchased by hospitals. There are also legislative opportunities; New York State passed a bill in late 2019 requiring a vegetarian option on the menu at every hospital.^50^ At one system in New York City, a gradual shift from 2019 to 2022 resulted in the default meal options at every public hospital to be a meatless one; 50%–60% of their patients just eat the default meal.^51^

Sustainability efforts involving food also include donating excess usable food and composting, which both save methane emissions. The Wellstar Health System in metropolitan Atlanta redistributes excess consumable food to needy individuals. St. Lawrence Health hospitals partner with a local farm on a composting initiative that keeps food waste out of landfills and allows this farm to produce natural fertilizer, thus doubly impacting climate benefits with less landfill emissions and less use of potentially environmentally damaging fertilizer products.

## Model and discuss the environmental and other benefits of diagnostic stewardship (not just ID tests!)

Diagnostic stewardship of microbiologic testing has been discussed in recent years and shows promise to both improve clinical care and decrease unnecessary antibiotics. ID professionals can expand this diagnostic stewardship to more routine laboratory testing, such as blood counts and inflammatory markers, such as C reactive protein. In Australia, it was estimated that basic hematology testing requires the energy equivalent of driving 0.4 miles per test.^52^ Radiologic testing, especially MRI is associated with high amounts of GHG emissions^53^, so stewardship of this type of testing should also be scrutinized with consideration of lower GHG testing, whenever possible, such as ultrasound.^52^ Finally, ID clinicians often generate extensive differential diagnoses, which is encouraged and should be maintained. However, the utility of ordering each diagnostic test should be scrutinized. The pretest probability of a diagnosis, the turnaround time available in relation to other testing that may be higher on the differential diagnosis, and the expected impact on clinical management should all be considered. Just by delaying some tests by 24–48 hours when there are more data available supports diagnostic stewardship and can decrease unnecessary healthcare resource use.

## Keep up your good work, and now start measuring the environmental benefits

Infectious diseases provide high-value care and focus on the prevention of illness. Many of our efforts are focused on decreasing hospitalization length of stay and appropriate outpatient parenteral treatment. Decreasing or preventing a hospital stay by just 1 day is estimated to save at least 45 kg of CO_2_ (equivalent to 115 miles driven by an average gas-powered vehicle).

If you are involved in quality improvement projects, consider adding environmental savings measurements to your work. This could be transportation-related travel savings (using the EPA calculator^54^), GHG emissions from solid waste in your health center (using a free online calculator),^55,56^ or GHG savings from reduced hospitalization length of stay in your existing work. The Centre for Sustainable Healthcare in the United Kingdom recognizes that healthcare value is best when patients have good clinical outcomes from a healthcare delivery system that generates the least harmful environmental, social, and financial impacts. There are many inspiring quality improvement projects that have been done by teams to help both patients and the planet.^57^

We recognize that helping to mitigate climate change can be overwhelming, but incorporating change in daily practices can lead to substantial benefits. If you would like to join other ID professionals to collaborate to decrease GHG emissions, email our group “Sustainabil-ID” at sustainabilityiddocs@gmail.com.

## Data Availability

Review article—not applicable.
